# A tail of two phages: genomic and functional analysis of *Listeria monocytogenes* phages vB_LmoS_188 and vB_LmoS_293 reveal the receptor-binding proteins involved in host specificity

**DOI:** 10.3389/fmicb.2015.01107

**Published:** 2015-10-09

**Authors:** Aidan Casey, Kieran Jordan, Horst Neve, Aidan Coffey, Olivia McAuliffe

**Affiliations:** ^1^Teagasc Food Research CentreFermoy, Ireland; ^2^Department of Biological Sciences, Cork Institute of TechnologyBishopstown, Ireland; ^3^Department of Microbiology and Biotechnology, Max Rubner-Institut, Federal Research Institute of Nutrition and FoodKiel, Germany

**Keywords:** *Listeria monocytogenes*, bacteriophages, *Siphoviridae*, serotype 4b/4e, comparative genomics, RBP

## Abstract

The physical characteristics of bacteriophages establish them as viable candidates for downstream development of pathogen detection assays and biocontrol measures. To utilize phages for such purposes, a detailed knowledge of their host interaction mechanisms is a prerequisite. There is currently a wealth of knowledge available concerning Gram-negative phage-host interaction, but little by comparison for Gram-positive phages and *Listeria* phages in particular. In this research, the lytic spectrum of two recently isolated *Listeria* monocytogenes phages (vB_LmoS_188 and vB_LmoS_293) was determined, and the genomic basis for their observed serotype 4b/4e host-specificity was investigated using comparative genomics. The late tail genes of these phages were identified to be highly conserved when compared to other serovar 4-specific *Listeria* phages. Spontaneous mutants of each of these phages with broadened host specificities were generated. Their late tail gene sequences were compared with their wild-type counterparts resulting in the putative identification of the products of ORF 19 of vB_LmoS_188 and ORF 20 of vB_LmoS_293 as the receptor binding proteins of these phages. The research findings also indicate that conserved baseplate architectures and host interaction mechanisms exist for *Listeria* siphoviruses with differing host-specificities, and further contribute to the current knowledge of phage-host interactions with regard to *Listeria* phages.

## Introduction

Bacteriophages are the most abundant entities in the biosphere, and play a pivotal role in contributing to the diversity of many microbial ecosystems through regular interaction with their bacterial hosts (Weitz et al., 2013). These phage-host interactions are primarily dependent upon the recognition of a bacterial cell wall constituent by a highly specific sequence on the phage surface known as the receptor binding protein. Other external factors may also dictate the nature of phage-host interactions, such as the temperature and pH of a given environment, which have been demonstrated to directly affect the efficiency of phage adsorption to a bacterial cell surface (Mahony et al., 2012). In general, a relatively high variability of bacterial host specificity exists amongst phages (Koskella and Meaden, 2013). Some phages exhibit the ability to infect a broad spectrum of strains within a bacterial genus (Carlton et al., 2005), while others are restricted to an extremely narrow host range consisting of a single strain within a bacterial subspecies (Bigby and Kropinski, 1989).

In the initial step of infection, phages adsorb to the surface of their bacterial host via the aforementioned interaction of a receptor binding protein (RBP) on the phage with a cell surface receptor on the host. The location and type of bacterial cell surface receptor can vary greatly, from cell wall teichoic acids and lipopolysaccharides to flagellar proteins (Wendlinger et al., 1996; Chaturongakul and Ounjai, 2014; Fokine and Rossmann, 2014), while RBPs generally have a conserved location at the distal end of the phage tail (Spinelli et al., 2006a). The host interaction mechanisms of many Gram-negative phages are well-established, particularly in the case of the *E. coli* myovirus T4 and the siphovirus lambda (Kostyuchenko et al., 2003; Leiman et al., 2003; Pell et al., 2009; Bartual et al., 2010; Chatterjee and Rothenberg, 2012; Fokine and Rossmann, 2014). As a member of the *Myoviridae*, T4 has a large contractile tail, terminating in a baseplate to which six long tail fibers are attached (Bartual et al., 2010). These fibers (encoded by genes *gp34-37*) are required for initial recognition and reversible binding of the host's cell-surface lipopolysaccharides (LPS), which is followed by signal transduction and extension of six shorter tail fibers from the baseplate that bind to the LPS irreversibly (Fokine and Rossmann, 2014). The short tail fibers are composed of homo-trimers of the gp12 protein, and represent the RBPs of phage T4 (Thomassen et al., 2003). A highly similar host interaction mechanism is observed in other Gram-negative *Myoviridae* such as the product of gene H in *E. coli* phage P2 (Haggård-Ljungquist et al., 1992) and the product of ORF69 and ORF84 in *Pseudomonas aeruginosa* phages PaP1 and JG004, respectively (Le et al., 2013). Homologous baseplate architectures to that of phage T4 have also been reported in a number of Gram-positive *Myoviridae* such as the *Bacillus* phage SPO1 and the *Staphylococcus* phage Twort (Habann et al., 2014). Bacteriophage lambda on the other hand is a member of the *Siphoviridae* that has a non-contractile tail, and adsorbs to its *E. coli* host via the highly specific irreversible interaction of its tail tip protein gpJ with the bacterial cell-surface maltose pore protein LamB (Chatterjee and Rothenberg, 2012; Samson et al., 2013). In contrast, the mechanisms of host-interaction and adsorption of most Gram-positive phages are relatively poorly understood. An exception to this are studies on the phages of the lactic acid bacteria (LAB) (Deveau et al., 2006; Spinelli et al., 2006b, 2014; Tremblay et al., 2006; Mahony and van Sinderen, 2014), particularly the lactococcal phages TP901-1 and Tuc2009 (Vegge et al., 2006). Phage TP901-1 interacts with its host using a baseplate that consists of two hexameric disk-like structures composed of trimers of the BppU and BppL (receptor binding) proteins (encoded by ORF48 and ORF49) (Veesler et al., 2012), while a homologous baseplate architecture has also been proposed for phage Tuc2009 (Sciara et al., 2008). The RBP of the *L. lactis* phage p2 (encoded by ORF18) has a different structural arrangement consisting of a three-subunit homo-trimer located on the distal end of the tail tube (Spinelli et al., 2006b; Tremblay et al., 2006), and is orthologous to the RBP in phages of other bacteria, including the tail spike protein gp21 of the *B. subtilis* phage SPP1 (Vinga et al., 2012), and indeed the gp12 protein of the Gram-negative *E. coli* phage T4 (Spinelli et al., 2006b).

*Listeria monocytogenes* is a bacterial pathogen that is the causative agent of listeriosis; a relatively rare yet severe disease that is associated with a mortality rate of 20–30%, and particularly affects elderly and immunocompromised individuals (Vázquez-Boland et al., 2001). The vast majority of reported clinical listeriosis cases arise following the consumption of contaminated food produce (Swaminathan and Gerner-Smidt, 2007), with *L. monocytogenes* strains of the 1/2a, 1/2b, 1/2c, and 4b serotypes being responsible for approximately 95% of all listeriosis outbreaks (Khen et al., 2015). Many phages infecting *L. monocytogenes* have been isolated and characterized (Carlton et al., 2005; Kim et al., 2008; Guenther et al., 2009; Vongkamjan et al., 2012) but only a limited number have been sequenced at the genome level (29 as of May 2015), and fewer still have been characterized in relation to their mechanisms of host interaction. An exception is the *L. monocytogenes* myovirus A511, where detailed transmission electron microscopy and immunogold-labeling experimentation has resulted in the identification of gp108 as the specific receptor binding protein and has led to the proposal of a model for adsorption for the SPO1-related phages (Habann et al., 2014). The receptor binding proteins in phage A511 manifest as short tail fibers located at the periphery of the tail when in its extended state, and following host binding, the tail contracts and these fibers are rearranged to the bottom of the baseplate (Habann et al., 2014). A model for the tail tip of the *Listeria* siphovirus A118 has also recently been proposed, and suggests a similar conformation to that seen in the *L. lactis* phage TP901-1, utilizing two hexameric disk-like structures composed of the gp19 and gp20 proteins in the recognition of and adsorption to its host (Bielmann et al., 2015).

Recent research by our group resulted in the isolation and whole genome sequencing of two phages, vB_LmoS_188 and vB_LmoS_293, which appeared to exhibit a host-specificity for *L. monocytogenes* strains of the 4b and 4e serotypes (Casey et al., 2015). The aim of this research was to determine the basis for this specificity through comparative genome, morphological, and functional analysis, to identify the genes responsible for binding of these phages to their bacterial hosts, and ultimately to contribute to the current repository of knowledge concerning *Listeria* phage-host interaction.

## Materials and methods

### Bacterial strains and culture conditions

**Figure 1:**
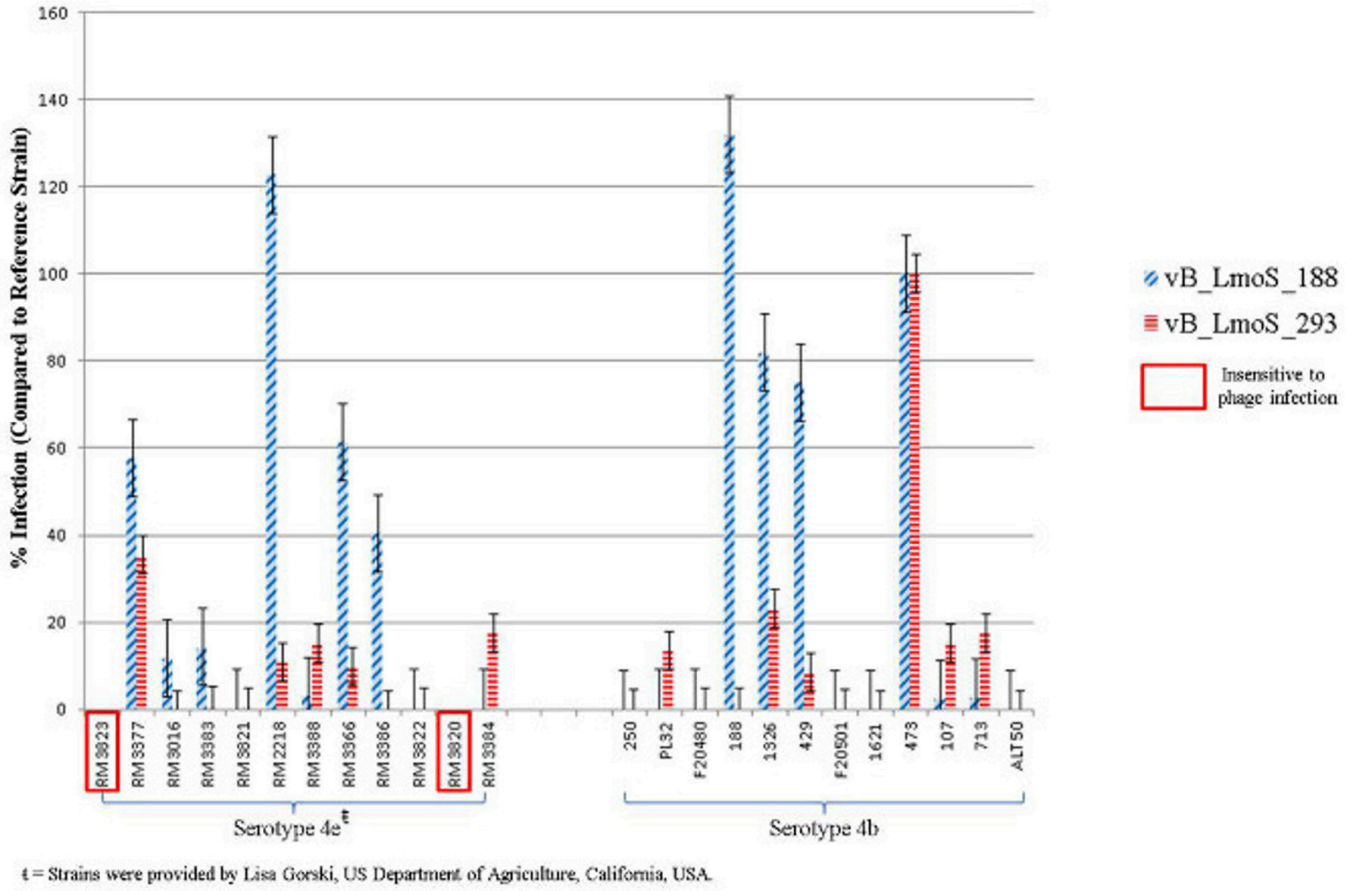
**Percentage infection levels of each of phages vB_LmoS_188 and vB_LmoS_293 against a selection of serotype 4b and 4e strains of *L. monocytogenes***. The percentages recorded are the average of duplicate assays, and have been normalized using strain 473 as a reference. The error bars for each value represent the standard error of the mean. Strains highlighted demonstrated no sensitivity to phage infection from either of the phages tested.

All *L. monocytogenes* strains used in this study were obtained from a collection of isolates housed at Teagasc Food Research Centre, Moorepark, unless otherwise indicated (Table 1, Figure 1). Overnight cultures of each strain were prepared following incubation in tryptic soy broth (TSB; BD Medical, Dublin, Ireland) at 37°C for 18 h under aerobic conditions. Solid media and soft agar overlays contained 1.5% and 0.7% agar, respectively.

**Table 1:** **Host range analysis of phages vB_LmoS_188, vB_LmoS_293 and the two phage mutants 293_Mut and 188_Mut generated in this study**.

**Bacterial Species**	**Host Strain**				Efficiency of plaquing^*^ of phages		
	**Strain no**.	**Serotype**	**Country of origin**	**vB_LmoS_293**	**vB_LmoS_188**	**293_Mut**	**188_Mut**
*Listeria monocytogenes*	PL38^±^	*1/2a*	Greece	0	0	0	0
	6179	*1/2a*	Ireland	0	0	0	0
	882	*1/2b*	Ireland	0	0	0	0
	1169	*1/2b*	Ireland	0	0	0	0
	6	*1/2c*	Ireland	0	0	0	0
	960	*1/2c*	Ireland	0	0	0	0
	34	*3a*	Ireland	0	0	0	0
	F2695^¥^	*4a*	USA	0	0	3.43 × 10^−1^	1.81 × 10^−1^
	SLCC661^†^	*4a*	Austria	0	0	0	0
	473	*4b*	Ireland	1	1	7.99 × 10^−1^	1
	429	*4b*	Ireland	5.3 × 10^−1^	4 × 10^−1^	4.64 × 10^−2^	2.17 × 10^−1^
	ALT50	*4b*	N.Ireland	7.88 × 10^−5^	9.46 × 10^−6^	1.13 × 10^−4^	2.11 × 10^−4^
	2944^†^	*4c*	Austria	0	0	7.86 × 10^−1^	2.17 × 10^−1^
	J2071^¥^	*4c*	USA	0	0	7.31 × 10^−2^	6.16 × 10^−2^
	33115	*4c*	Ireland	0	0	1	2.8 × 10^−1^
	R2517^¥^	*4d*	USA	0	0	0	0
	R9916^¥^	*4e*	USA	2.11 × 10^−4^	6.21 × 10^−5^	1.33 × 10^−5^	1.32 × 10^−4^
	CDL228^†^	*4e*	Austria	2.49 × 10^−4^	1.32 × 10^−4^	1.01 × 10^−5^	1.02 × 10^−4^
	R9915^¥^	*7*	USA	0	0	0	0
*Listeria innocua*	S4378^¥^	*4ab*	USA	0	0	0	0

The efficiency of plaquing (EOP) figures for each of the phages is presented.

* All results are the average of duplicate assays.

¥ Strains were provided by Prof. Martin Wiedmann, Cornell University, New York, USA.

† Strains were provided by Prof. Martin Wagner, University of Veterinary Medicine Vienna, Austria.

± Strain was provided by Dr. Panagiotis Skandamis, Agricultural University of Athens, Greece.

### Bacteriophage lytic spectrum determination

Bacteriophages vB_LmoS_188 and vB_LmoS_293 (hereafter referred to as M188 and MC293, respectively) were previously isolated from separate environmental sources (wild mushrooms for M188, mushroom compost for MC293) and propagated following previously defined protocols (Alemayehu et al., 2009; Cavanagh et al., 2013). The lytic spectrum of each of the phages was determined by plaque assay against a range of *L. monocytogenes* strains (Table 1). Briefly, for each of the phages, 1 ml of lysate (approximately 1 × 10^7^ plaque forming units (pfu)/ml for M188, 1 × 10^8^ pfu/ml for MC293) was serially diluted in 9 ml of maximum recovery diluent (MRD; Oxoid, Hampshire, UK). For each dilution, 1 ml of the serially diluted lysate was mixed with 100 μl of an overnight culture of a given test strain (10^7^ colony forming units (cfu) approximately) and 100 μl of 0.185 M CaCl_2_ in a test tube containing 5 ml TSA soft agar, which was then vortexed and plated onto a TSA plate. Plaque assay enumerations were calculated from the results of independent duplicate testing. Efficiency of plaquing (EOP) figures (Table 1) were calculated by dividing the phage titre (in pfu/ml) of any given test strain, by the phage titre (in pfu/ml) of the phage-sensitive indicator strain (the strain against which the greatest number of plaques were produced). For phages M188, MC293, and 188_Mut, the serotype 4b strain 473 was selected to be the phage-sensitive indicator, while the serotype 4c strain 33115 was chosen as the phage-sensitive indicator for phage 293_Mut.

### Adsorption assays

Relative levels of adsorption of both phages to cells of *L. monocytogenes* was determined as follows: For each of the phages assessed, 500 μl of lysate (approximately 5 × 10^6^ pfu of M188, 5 × 10^7^ pfu of MC293) was added to eppendorf tubes (Sarstedt) containing 1 ml aliquots of an overnight culture of each test strain (approximately 10^8^ cfu) and 100 μl of 0.185 M CaCl_2_. A control tube that contained no *Listeria* cells was also prepared. All tubes were incubated for 20 min at 37°C, before being centrifuged at 12,000 × g for 10 min at room temperature. Each of the samples was then passed through a 0.2 μm filter (Sarstedt, Co. Wexford, Ireland), with 1 ml of the resulting filtrate then serially diluted in MRD to a dilution of 10^−7^. All dilutions for each sample were subsequently plated (as described for the plaque assay assessments) against the sensitive serotype 4b host strain 473, and incubated overnight at 37°C. Adsorption assay enumerations were calculated from the results of independent triplicate testing. The % adsorption figures were calculated using the formula [((control titre–titre in supernatant)/control titre) × 100], where the control titre was the number of plaque forming units present in the control tube.

### Ammonium acetate precipitation of bacteriophages

Pure samples of each of the phages were obtained by ammonium acetate precipitation. A 1.2 L lysate of each of the phages was prepared, which was then centrifuged at 6000 × g for 20 min and passed through a 0.45 μm filter (Sarstedt) to remove bacterial cell debris. The filtrates were then centrifuged at 38,000 × g for 1 h at 4°C, following which the supernatant was discarded, and the pellet from each sample was resuspended in 10 ml ice-cold ammonium acetate (0.1 M). The samples were once more centrifuged at 38,000 × g for 1 h at 4°C, after which the pellets were resuspended again, this time in a final volume of 1 ml of 0.1 M ammonium acetate.

### Electron microscopy

Preparations of each of the phages from the ammonium acetate precipitation were negatively stained with 2% uranyl acetate on carbon films. Each grid was examined at an 80 kV acceleration voltage using a Tecnai 10 transmission electron microscope (FEI Company, Eindhoven, Netherlands). Micrograph images were captured using a MegaView 2 CCD-camera (Olympus SIS, Münster, Germany). Structure dimensions of each of the phages were determined based on the average of up to 20 measurements.

### Phage DNA extractions

Preparations of each of the phages from the ammonium acetate precipitation were subject to DNA extraction as follows: To each eppendorf tube (Sarstedt) containing 1 ml of phages in 0.1 M ammonium acetate, 5 μl of CaCl_2_ (0.185 M), 5 μl DNase I solution (Life Technologies, Co. Dublin, Ireland) and 5 μl RNase A (Life Technologies) were added, with samples subsequently incubated for 1 h at 37°C. Each sample was then centrifuged at 6200 × g for 10 min at 4°C, after which the supernatant was transferred to a sterile eppendorf tube. Next, 100 μl of SDS mix (containing 0.5 M Tris–HCl pH 9.0, 0.25 M EDTA, 2.5% SDS) was added to each tube, with samples incubated for 30 min at 65°C. Following this, 125 μl of 8 M potassium acetate (Sigma-Aldrich, Dublin, Ireland) was added. Samples were placed on ice for 30 min, and then centrifuged at 12,000 × g for 10 min. The supernatant (approximately 800 μl) was transferred to a sterile eppendorf tube, where an equal volume of phenol chloroform isoamyl alcohol (25:24:1) (Sigma) was added. The tubes were then centrifuged for 5 min at 12,000 × g. The top layer of the resulting sample (approximately 700 μl) was transferred to a sterile eppendorf tube and this step was repeated once more. Following the second phenol chloroform isoamyl alcohol wash step, the top layer (approximately 600 μl) was transferred to a sterile eppendorf tube, and an equal volume of ice-cold isopropanol (Sigma) was added. Samples were incubated at −20°C for 30 min, and then centrifuged at 12,000 × g for 20 min at 4°C. The supernatant was decanted, and the resulting pellet of DNA was washed twice with 200 μl of 70% ethanol (Sigma), and resuspended in 40 μl of PCR grade H_2_O (Medical supply company, Co. Dublin, Ireland).

### Comparative genomic analysis

Fully sequenced and assembled *Listeria* phage genomes used for comparison, including the sequences of M188 and MC293, were obtained from the NCBI database (http://www.ncbi.nlm.nih.gov/genome). Linear genome comparisons of these phages were undertaken using Easyfig (Sullivan et al., 2011). For each input phage in this analysis, the protein products of each coding sequence were concatenated into a single file. Progressive multiple protein sequence alignments were then performed *via* the slow-accurate ClustalW method using the Gonnet series protein weight matrices, and were generated in the MegAlign application of the Lasergene Genomics Suite (DNAStar Inc., Madison, USA). A phylogenetic tree of the alignment was generated in MegAlign using the Kimura distance formula, with bootstrap analysis performed using default parameters of trial number = 1000 and random seed number = 111. HHpred analyses of protein sequences were undertaken using the online bioinformatics toolkit software on the HHpred interactive server (http://toolkit.tuebingen.mpg.de/hhpred).

### Generation of spontaneous phage mutants

To generate spontaneous mutants of each of the phages, 1 ml of lysate (approximately 1 × 10^7^ pfu of M188, 1 × 10^8^ pfu of MC293) was added in each case to a sample tube containing 5 ml TSB, 400 μl of 0.185 M CaCl_2_, 200 μl of overnight culture (approximately 2 × 10^7^ cfu) from the sensitive serotype 4b host strain 473, and 600 μl of overnight culture (approximately 6 × 10^7^ cfu) from the insensitive serotype 4c host strain 33,115. The sample tubes were incubated at 37°C for 3 h, after which an additional 400 μl of overnight culture from the sensitive serotype 4b host was added to each, before returning the tubes to the incubator for a further 3 h at 37°C. The resulting samples were then filter-sterilized through a 0.45 μm filter (Sarstedt), after which the filtrates were plaqued, as previously described, against the insensitive serotype 4c host strain 33,115. Plaques observed on the resulting plates were then purified, propagated, and stocked as described previously (Cavanagh et al., 2013).

### Primer design and PCR amplification

Primer sequences (Table 2) were designed for the purpose of PCR analysis using the SeqBuilder application of the DNAStar Lasergene Genomics Suite (DNAStar), with the subsequently designed primers synthesized by Sigma-Aldrich (Sigma-Aldrich). Amplicons were generated using these primers, together with template bacteriophage DNA and Platinum Hi-fidelity PCR Supermix (Biosciences Ltd, Dun Laoghaire, Dublin), under the following PCR conditions: 95°C for 5 min followed by 40 cycles of 94°C for 1 min, 50°C for 15 s and 72°C for 1 min, with a final 72°C for 5 min step at the end. In order to check the quality of amplified DNA, 5 μl of each PCR sample was run on a 1% agarose gel for 60 min at 100 volts, with DNA subsequently visualized under UV light. Successfully amplified fragments of DNA were then sent for direct sequencing (GATC Biotech, Koln, Germany).

**Table 2:** **Primers sequences designed for DNA amplification of the late tail genes in phages 188_Mut and 293_Mut**.

**Primer name**	**F/R**	**Genomic region/ORF amplified**	**Primer sequence**
1881819F1	F	Six sets of overlapping forward and reverse primers covering phage vB_LmoS_188 ORFs 18&19	5′-tatcgaaaacattggcac-3′
1881819R1	R		3′-agctttttgaagtggttt-5′
1881819F2	F		5′-gatgattctactactacagc-3′
1881819R2	R		3′-ataatcacatcttcatcata-5′
1881819F3	F		5′-aagatagattagatagtgac-3′
1881819R3	R		3′-taagtctagctgtcccgcct-5′
1881819F4	F		5′-gcgatagacccgtttaca-3′
1881819R4	R		3′-agttagttttatctgctg-5′
1881819F5	F		5′-agcattaatagatagcga-3′
1881819R5	R		3′-tacattaaaaactggatt-5′
1881819F6	F		5′-ttttcaatctcaatagcggt-3′
1881819R6	R		3′-tccaaaacaattaagtcgtc-5′
188202122F1	F	Forward and reverse primers covering phage	5′-atattgttggtgttggct-3′
188202122R1	R	vB_LmoS_188 ORFs 20, 21, and 22	3′-tcagatatagtaaagcct-5′
29319F1	F	Forward and reverse primers covering phage	5′-ttacaaacaaaccaccagaa-3′
29319R1	R	vB_LmoS_293 ORF 19	3′-cagcagacgataatactaaa-5′
29320F1	F	Forward and reverse primers covering phage	5′-ttttcaatctcaatagcggt-3′
29320R1	R	vB_LmoS_293 ORF 20	3′-tccaaaacaattaagtcgtc-5′
293212223F1	F	Forward and reverse primers covering phage	5′-atattgttggtgttggct-3′
293212223R1	R	vB_LmoS_293 ORFs 21, 22, and 23	3′-tcagatatagtaaagcct-5′

## Results

### Both phages exhibited a host-specificity for serotype 4b and 4e strains

**Figure 2:**
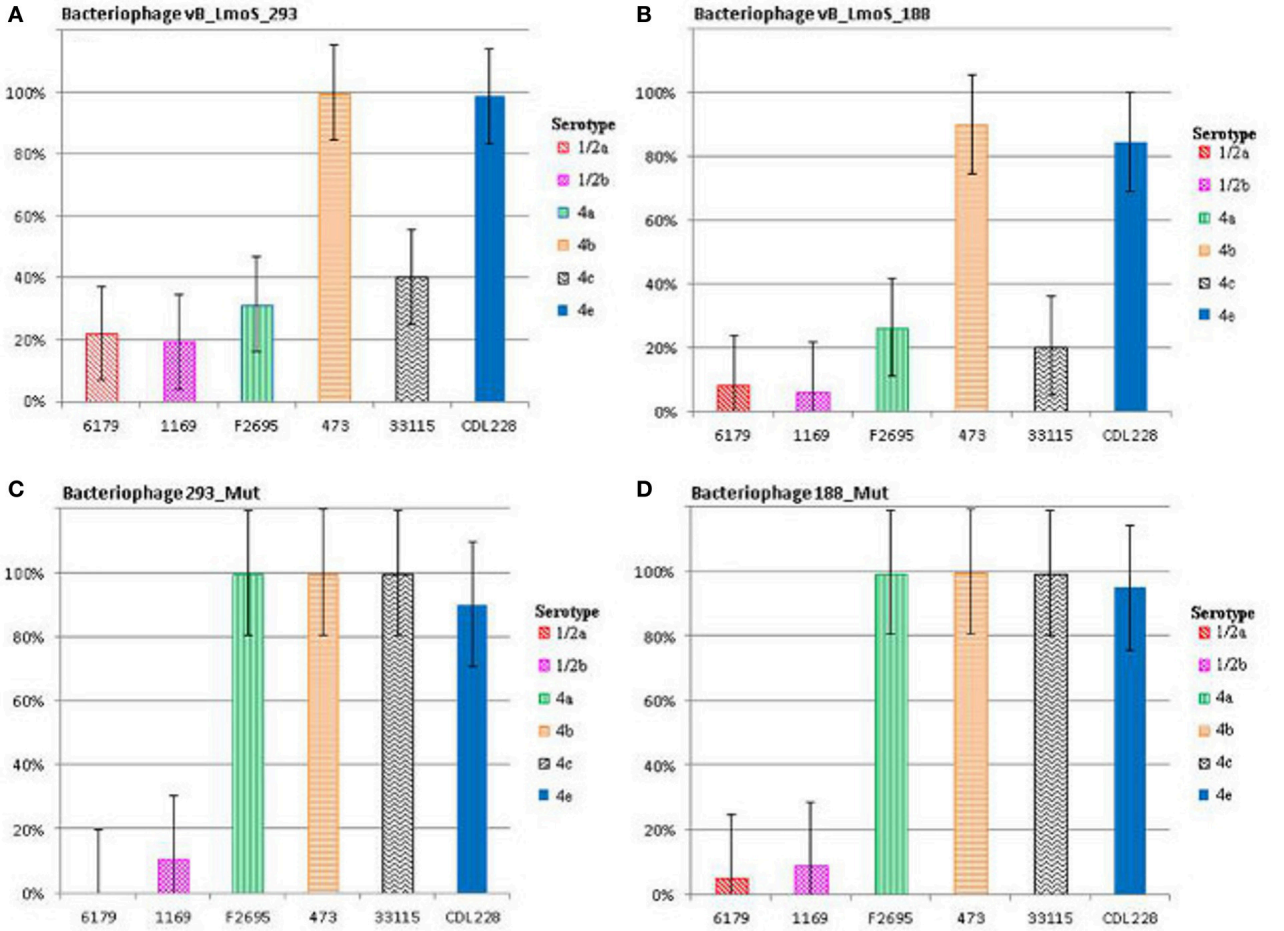
**Relative % adsorption of phages against a selection of *L. monocytogenes* strains of differing serotypes; vB_LmoS_293 (A), vB_LmoS_188 (B), 293_Mut (C), 188_Mut (D)**. The numbers are the average of triplicate tests, and represent the percentage of phage particles adsorbed to the surface of each test strain compared to the control. The error bars for each value represent the standard error of the mean.

The lytic spectrum of phages vB_LmoS_188 and vB_LmoS_293 (hereafter referred to as M188 and MC293, respectively) was determined by plaque assay against a number of *L. monocytogenes* strains. The strains selected for analysis represented isolates from a range of environmental niches, sample types, serotypes, and geographic locations (including the Republic of Ireland, Northern Ireland, USA, Austria, and Greece). The results of the plaque assay assessments (Table 1) revealed that phages M188 and MC293 have a relatively narrow specificity in terms of host range. Both of the phages were shown to be capable of infecting 4b and 4e strains of *L. monocytogenes*. None of the other serotypes tested (including 1/2a, 1/2b, 1/2c, 3a, 4a, 4c, 4d, and 7) exhibited any degree of sensitivity to infection with M188 and MC293. These observations were subsequently confirmed through additional testing against a larger selection of *L. monocytogenes* serotype 4b and 4e isolates, all of which were sensitive to both phages, with the exception of the serotype 4e strains RM3820 and RM3823 (Figure 1). Subsequent adsorption assays demonstrated that the levels of adsorption of MC293 to the serotype 4b and 4e strains were >95% compared to <40% for any of the other serotypes tested (Figure 2A). Similar observations were noted for M188, where levels of adsorption to the serotype 4b and 4e strains were >80% compared to <30% for the other serotypes assessed (Figure 2B).

### Phages M188 and MC293 belong to *Listeria* phage orthocluster IV

**Figure 3:**
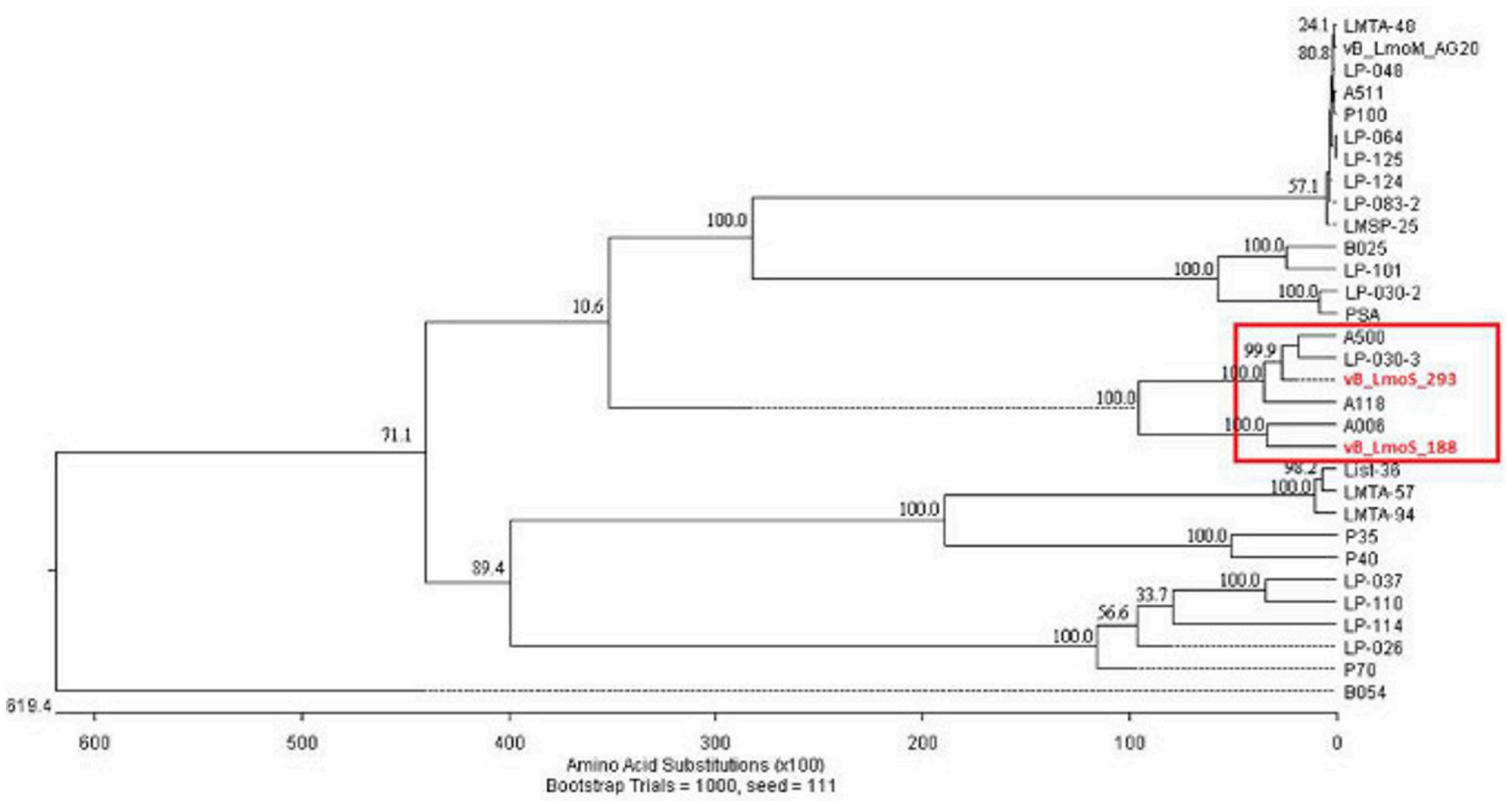
**Phylogenetic analysis of phages vB_LmoS_188, vB_LmoS_293 and other sequenced *L. monocytogenes* bacteriophages**. The tree was generated in MegAlign (DNAStar); input sequences for ClustalW alignment consisted of whole proteomes of each bacteriophage.

**Figure 4:**
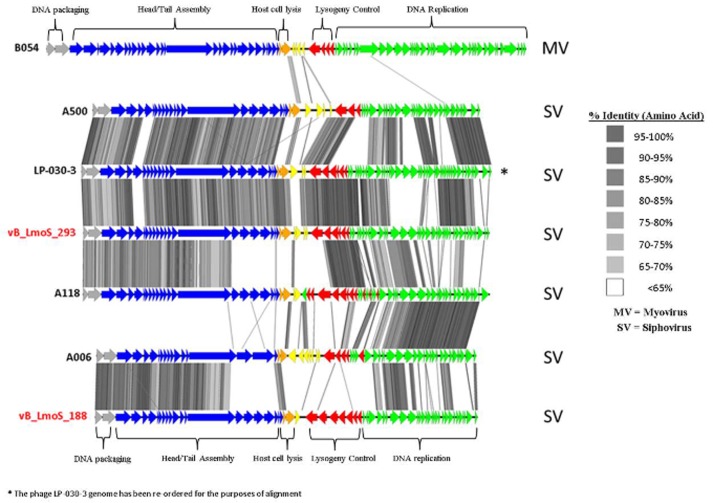
**Genome comparisons (amino acid level) of *L. monocytogenes* phages of Orthocluster IV, visualized in EasyFig**.

As a result of whole proteome multiple sequence alignments, phages M188 and MC293 were determined to cluster with the temperate phages A500, A118, A006, and LP-030-3 (Figure 3), in a previously defined group of *L. monocytogenes* phages denoted as Orthocluster IV (Denes et al., 2014). The clustering approach employed for this research differed from that of Denes et al., with concatenated protein products from each phage utilized in multiple protein sequence alignments in this study, whereas Denes et al clustered the *Listeria* phages on the basis of the presence/absence of orthologous genes with particular BLAST and % MCL cutoffs used for defining orthology. A comparison of the tree generated in this study with the Orthoclusters defined by Denes et al identified extensive similarities, as the phages belonging to each Orthocluster in the previous study were shown to cluster together in the tree generated from this research. Additionally, *Listeria* phage B054 was identified to be an outlier in both studies. Analogous to other phages within Orthocluster IV, M188 and MC293 also harbored genomes of between 38 and 41 kbp in size, containing 62–73 genes, with a G+C content of 35.5–36.5%. The Orthocluster IV phages were then subject to linear comparison in order to determine the extent of their genetic relatedness (Figure 4). Each of the six genomes within this Orthocluster demonstrated a modular structure that is consistent with temperate phages (Loessner et al., 2000). The genomes can be described as being composed of five distinct and ordered gene clusters, with general functions in DNA packaging, structural assembly, host cell lysis, lysogenic conversion, and DNA replication/modification. Linear comparisons revealed that phages A500, A118, LP-030-3, and MC293 formed a more closely related group within this Orthocluster, with phages M188 and A006 grouping together within a separate subcluster. The closest relative to phage MC293 was identified to be phage LP-030-3 (Denes et al., 2014), as these two phages exhibited an overall amino acid sequence identity of 91% across 85% of their respective genomes. The high degree of homology observed between these two phages was particularly evident in their respective lysogeny control and host cell lysis gene clusters, as well as in their structural assembly modules. Virtually no homology between these two phages was observed in the amino acid sequences of their respective main capsid proteins. Instead, the main capsid protein of phage MC293 was determined to be most closely related to that of phage A118, with 92% identity observed between the protein sequences of each. Phage M188 and its closest relative phage A006 (Dorscht et al., 2009) shared an amino acid identity of 90% across 65% of their respective genomes, particularly in their DNA packaging and phage head assembly proteins. However, no homology was identified between their late tail proteins or their lysogenic conversion modules, suggesting these phages may utilize differing methods of host infection.

### Comparative genome analysis reveals underlying conservation in phage tail sequences

**Figure 5:**
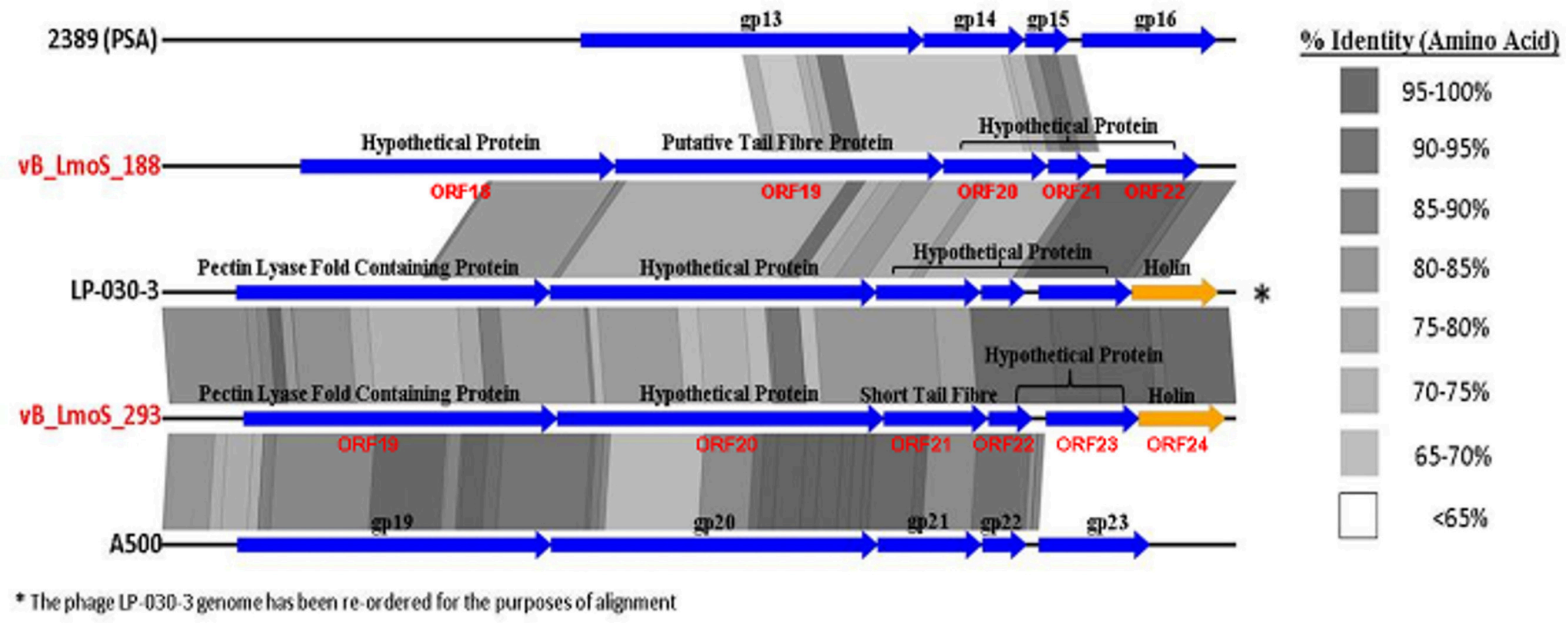
**A close comparison of the late tail genes from five serovar 4-specific phage genomes, labeled with their protein products**.

The host-specificity of each of the input phages for this analysis was recorded where possible, and phages with a similar lytic spectrum to that observed for phages M188 and MC293 were selected for closer comparative analysis. Two of the Orthocluster IV phages, namely LP-030-3 (Denes et al., 2014), and A500 (Dorscht et al., 2009) were selected for analysis, given their specificity for serovar 4 strains of *L. monocytogenes*, as was phage 2389 (PSA) (Zimmer et al., 2003) which is a serovar 4-specific member of the Orthocluster III phages, and displays little overall homology to phages M188 or MC293. The other sequenced phages used in this study either had undefined or vastly different host-specificities to these five phages, including the other members of Orthocluster IV, namely phages A006 and A118, which both have a serovar 1/2 host-specificity. The results of this comparison (Figure 5) identified a region of highly conserved amino acid sequence across all five genomes, located specifically between the genes encoding the tail tape measure protein and the host cell lysis machinery of each phage. As such, the open reading frames (ORFs) highlighted by this comparison were selected for further investigation. Translated protein products from these ORFs (specifically ORFs 18–22 in M188 and ORFs 19–23 in MC293) were subjected to HHpred analyses (Söding et al., 2005) in order to identify structural homologs in other phages. Homology to other phage-related proteins could not be identified in the case of the translated protein products of ORFs 18, 20, 21, and 22 in phage M188, or in the case of the products of ORFs 19, 21, 22, and 23 in phage MC293. However, the products of ORF 19 in phage M188 and ORF 20 in phage MC293 demonstrated N- and C-terminal structural homologies to the products of ORFs 48 and 49 of the *Lactococcus* phage TP901-1, which encode the BppU (baseplate protein upper) and BppL (baseplate protein lower / receptor binding) proteins of this phage, respectively.

### Both phages belong to morphospecies 2671 of the *Listeria siphoviridae*

**Figure 6:**
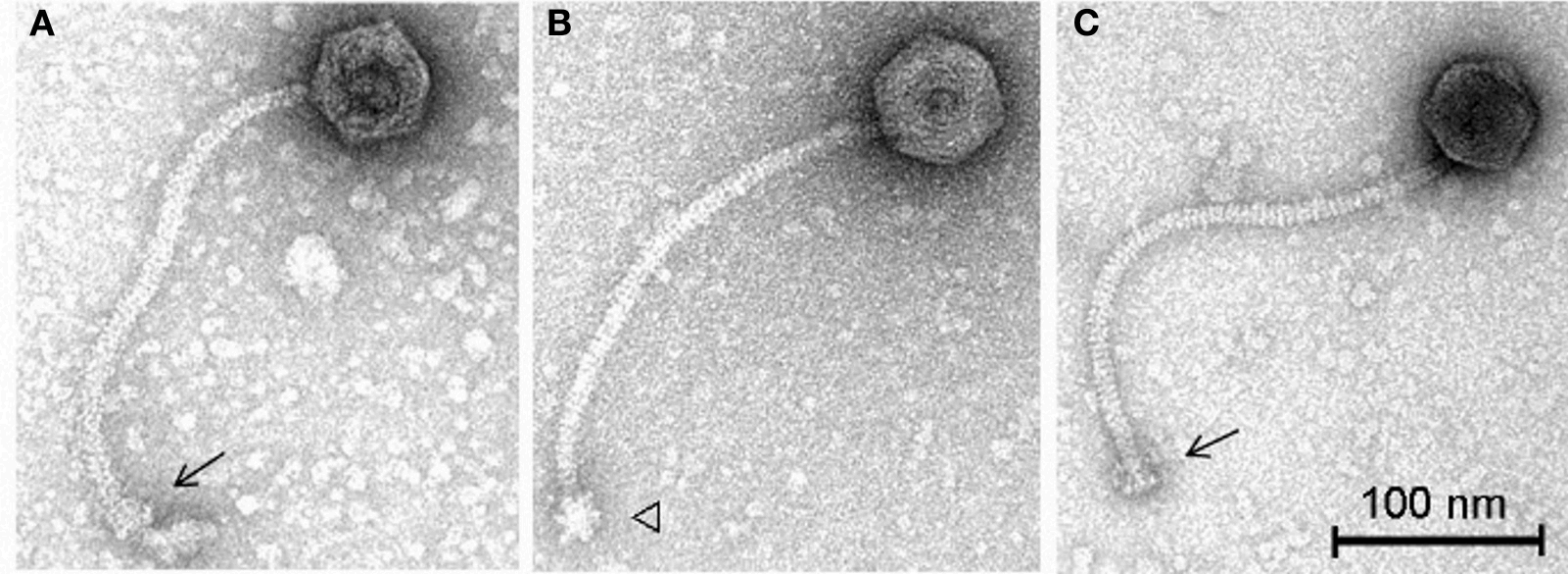
**Transmission electron micrographs of phages vB_LmoS_188 (A) and vB_LmoS_293 (B,C) negatively stained using 2% uranyl acetate**. The double-discs conformation of the baseplates is indicated by the arrows **(A,C)**. The six-pointed star conformation of the phage vB_LmoS_293 baseplate is indicated by the triangle in **(B)**.

To further characterize these phages in terms of morphology, electron microscopy analysis was performed. Phage vB_LmoS_188 was determined to possess an isometric capsid of 59.3 ± 3.9 nm (*n* = 20) in diameter with a tail approximately 261.5 ± 7.5 nm (*n* = 20) in length (Figure 6A), while phage vB_LmoS_293 was slightly larger, with a capsid diameter of 60.0 ± 3.5 nm (*n* = 17) and tail length of 278.6 ± 13.1 nm (*n* = 16) (Figures 6B,C). Given their physical structure, both phages were classified as *Siphoviridae*. Furthermore, the two phages were additionally classified as members of morphospecies 2671 of the *Listeria* phages, due to their characteristically long and flexible, transversely striated tails, terminating with typical double-discs baseplates (Figures 6A,C). For phage vB_LmoS_293, bottom views of the baseplates were frequently detected with tail spikes that manifest themselves in the shape of a six-pointed star (Figure 6B) (Ackermann and DuBow, 1987; Denes et al., 2014) with a diameter of 23.1 ± 2.1 nm (*n* = 7). This hexameric conformation of the vB_LmoS_293 phage baseplate is comparable to previous observations for phages LP-030-3 (Denes et al., 2014) and A118 (Bielmann et al., 2015). A similar conformation was not observed in the case of phage vB_LmoS_188. However, this phage is presumed to have an equivalent baseplate structure, given the high degree of conservation that was observed between the late tail genes of this phage and those of phages LP-030-3 and vB_LmoS_293.

### Phage mutants exhibited enhanced serovar 4 host-specificities

A spontaneous mutant of each of phages M188 and M293 (denoted here as 188_Mut and 293_Mut, respectively) was generated following exposure to an insensitive serotype 4c host strain of *L. monocytogenes*. The lytic spectrum of each of the resulting mutants was determined against the same selection of *L. monocytogenes* isolates used in the initial host range testing of phages M188 and MC293. The results demonstrated that both phages 188_Mut and 293_Mut were capable of infecting a number of serotype 4c strains of *L. monocytogenes* and a single serotype 4a strain (F2695), while still retaining their original infection abilities (Table 1). Additionally, the EOP figures calculated for both of the mutants indicated that they were able to infect the new serotype 4c host strains with a similar efficiency to the serotype 4b host strains. Furthermore, the relative level of adsorption of phages 293_Mut (Figure 2C) and 188_Mut (Figure 2D) to all of the serovar 4 strains assessed was identified to be >90% compared to figures of <20% for both of the tested serotype 1/2a and 1/2b strains, respectively.

### Mutations in a specific late tail gene are implicated in broadening host-specificity

**Figure 7:**
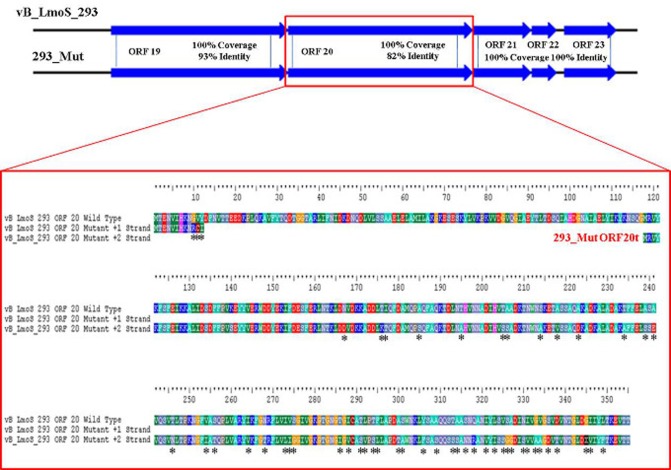
**Amino acid sequence alignment of open reading frame (ORF) 20 of phage vB_LmoS_293 and its corresponding ORF in phage 293_Mut**. Alterations in amino acid sequence in the mutant ORF compared to the wild-type are denoted with asterisks.

**Figure 8:**
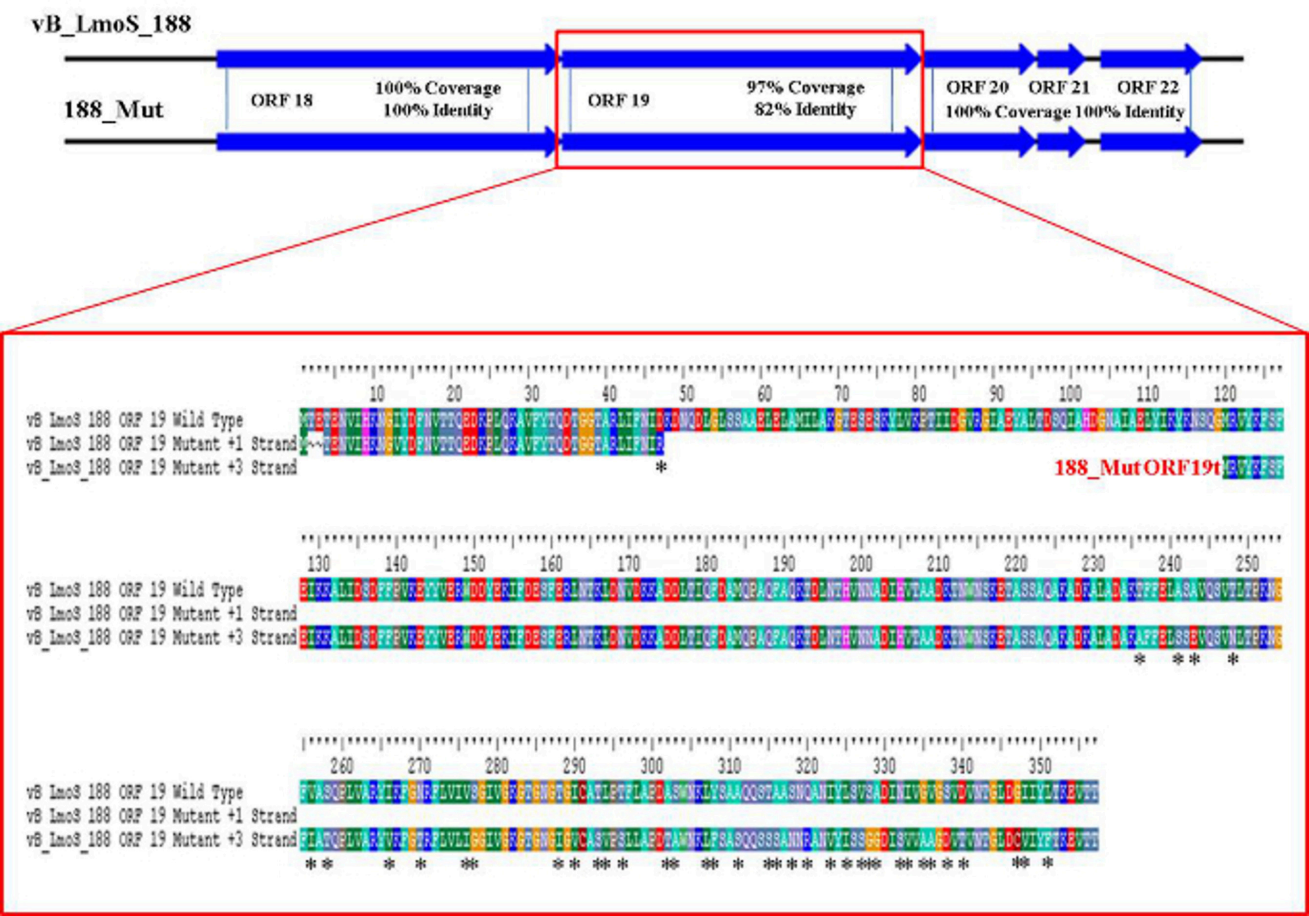
**Amino acid sequence alignment of open reading frame (ORF) 19 in phage vB_LmoS_188 and its corresponding ORF in phage 188_Mut**. Alterations in amino acid sequence in the mutant ORF compared to the wild-type are denoted with asterisks.

As mentioned previously, a region containing five ORFs in phages M188 and MC293 is thought to be responsible for bacterial host-specificity. The sequence of this five-gene region was examined in both mutants. Three of these ORFs in the 293_Mut mutant (ORFs 21, 22, and 23) were determined to be 100% identical to the corresponding ORFs in the wild-type phage MC293. The translated protein product from ORF 19 of the 293_Mut genome differed from the wild-type phage at two locations; a change from a phenylalanine to a leucine at residue 3, and a change from a threonine to an isoleucine at residue 241. Comparison of ORF 20 in the 293_Mut genome with the corresponding ORF in the wild-type phage, however, revealed that this particular gene had undergone extensive mutation during the generation of phage 293_Mut (Figure 7), with these two ORFs sharing just 82% nucleotide sequence identity. In terms of a translated protein product, this particular ORF in phage 293_Mut putatively encoded a protein of 238 amino acids in length (denoted 293_Mut ORF20t), differing in composition to the corresponding region in the wild type phage MC293 at 49 of the 238 residues. Subsequent HHpred analysis of this hypothetical protein product revealed that it still retained structural homology to the aforementioned *Lactococcus* phage TP901-1 receptor binding protein (BppL), despite the numerous amino acid mutations. A linear comparison of the amplified region of the 188_Mut genome with that of phage M188 revealed that the majority of the ORFs in this cluster remained unchanged in the mutant when compared to the wild-type, with the exception of ORF 19 (Figure 8). Like its counterpart in phage 293_Mut, ORF 19 in phage 188_Mut also underwent extensive mutation during the generation of this mutant. This ORF was also determined to putatively encode a 238 amino acid protein product (denoted 188_Mut ORF19t), which differed in composition to the corresponding region in the wild-type phage at 39 of the 238 residues. HHpred analysis similarly revealed retention of structural homology to the BppL receptor binding protein of phage TP901-1 for this hypothetical protein product.

## Discussion

This research focused on identifying the underlying genomic basis for the perceived host-specificities of phages M188 and MC293, and to provide a further insight into the complex mechanisms of phage-host interaction in *Listeria* phages in general. Initial lytic spectrum assessments established that both phages M188 and MC293 shared a strict specificity for *L. monocytogenes* strains of the 4b and 4e serotypes. Interestingly, two of the serotype 4e strains tested (RM3820 and RM3823) were observed to be completely insensitive to infection from either of these two phages. While the precise genetic basis for this insensitivity is unknown, it is possible that these particular strains may harbor additional internal resistance mechanisms such as CRISPR/Cas or restriction modification systems, which can act as secondary defenses to phage infection through recognition and cleaving of injected phage DNA (Orsi et al., 2011; Samson et al., 2013). Additional resistance mechanisms may also have been present in some of the other serotype 4b and 4e host strains, which had low calculated EOP figures (Table 1) despite the relatively high adsorption levels to serotype 4b and 4e host cell surfaces that were observed for each of the phages (Figures 2A,B). Likewise, upwards of 20% adsorption was observed for particles of phage MC293 to the cell surface of the tested insensitive serotype 1/2a and 1/2b strains, while 40% adsorption was observed for particles of phage MC293 to the cell surface of a tested insensitive serotype 4c host strain (Figure 2A). However, phage MC293 was experimentally determined to be incapable of establishing an infection in any of these three host strains, suggesting that similar secondary bacterial host resistance mechanisms may also have been present in these insensitive host strains. Limited host range analysis outside of the *L. monocytogenes* species indicated that neither of the phages were capable of infecting a serotype 4ab strain of *L. innocua*, but that both phages could infect strain S4-120 of *L. marthii* (Graves et al., 2010). Recent research has demonstrated that the *L. monocytogenes* and *L. marthii* species are closely related to one another phylogenetically, with *L. marthii* described as forming a “sister group” to *L. monocytogenes* (den Bakker et al., 2010). Therefore, the ability of phages MC293 and M188 to infect this strain could have been made possible by the evolutionary conservation of cell wall constituents between these *Listeria* species. Sensitivity of *L. marthii* to a *Listeria* phage has been previously reported in the case of the unique virulent *L. monocytogenes* phage P70 (Schmuki et al., 2012), though virtually no sequence homology exists between P70 and the two phages isolated in this study, suggesting that they employ contrasting mechanisms for host binding and infection. No specific phages of *L. marthii* have been isolated to date (Klumpp and Loessner, 2013). The results of these host range analyses demonstrate the potential for exploiting the genetic components of phages M188 and MC293 for a number of downstream applications. In the past, the biologically relevant properties of phages have revealed their potential for use in the formulation of biocontrol products (Leverentz et al., 2003; Carlton et al., 2005; Mahony et al., 2011), as well as in the development of rapid pathogen detection assays, through the isolation and fluorescent tagging of cell binding domains (CBDs) from phage tail proteins (Hagens and Loessner, 2007; Smartt et al., 2012). Due to their narrow perceived host specificities, phages M188 and MC293 could similarly be adapted in the development of specialized serotyping kits for *L. monocytogenes* strains of the 4b and 4e serotypes. A rapid and accurate method for detecting the presence of serotype 4b/4e isolates of *L. monocytogenes* within a sample could be of particular benefit to areas such as the food processing industry, given their clinical relevance and known association with listeriosis outbreaks.

Comparative genomic analysis involving *Listeria* phages with similar host-specificities to M188 and MC293 highlighted a region of sequence conservation across their respective genomes. Prior research had indicated that this region was associated with encoding the phage baseplate and host-recognition components of these types of phages (Le et al., 2013; Marti et al., 2013; Stockdale et al., 2013; Bielmann et al., 2015), and as such, the genes within this region were selected as candidates for the RBP of phages M188 and MC293. Spontaneous mutants of each of phages M188 and MC293 with broadened host-specificities to include *L. monocytogenes* serotypes 4a and 4c were generated, and the candidate RBPs in these mutants were sequenced for comparison with their wild-type counterparts. A similar technique was also employed in an attempt to generate a serovar 1/2-specific mutant of each of these phages, but these assays proved unsuccessful. This is likely due to the fact that the sugar substituents in *L. monocytogenes* cell wall teichoic acids are serovar-specific (Loessner et al., 2002). As such, in order for the mutants to become serovar 1/2-specific, the late tail genes of these phages would need to undergo far greater a level of alteration than what is achievable by spontaneous mutation alone. The enhanced host-specificities in both mutant phages were observed to be associated with alterations in one particular ORF in each of their genomes (corresponding to ORF 19 in M188 and ORF 20 in MC293). HHpred analyses identified structural homologies between the N- and C-terminal products of these ORFs in the wild-type phages and the BppU and BppL (receptor binding) proteins of the *Lactococcus* phage TP901-1 (Veesler et al., 2012). These structural homologies, coupled with the sequence alterations in their respective mutant phages, led to the putative identification of the protein products of ORF 19 in M188 and ORF 20 in MC293 as the RBPs of these phages. The results correlated with similar observations for gp20 of the *Listeria* phage A118, which has recently been identified as the receptor binding protein of this particular phage, and which also demonstrates N- and C-terminal structural homologies with the BppU and BppL proteins of phage TP901-1 (Bielmann et al., 2015). In addition, TEM imaging of phage MC293 revealed it to have a hexameric baseplate architecture, which is also homologous in conformation to the baseplate structures previously described for both of phages A118 (Bielmann et al., 2015; Cambillau, 2015) and TP901-1 (Veesler et al., 2012). Subsequent research involving gp20 of phage A118 has suggested that this protein in fact represents a fusion between functional analogs of the BppU and BppL proteins in phage TP901-1, and that the baseplate of phage A118 may therefore have arisen from a mutation event in a lactococcal phage that resulted in an altered ability to recognize the cell surface saccharides of the *Listeria* species (Cambillau, 2015). As such, it is conceivable to propose that a similar evolutionary event may have resulted in the emergence of phages M188 and MC293. However, in-depth characterization of the M188 and MC293 baseplates would be required in order to fully determine the extent of their relationship with those of TP901-1 and A118. Interestingly, in terms of amino acid sequence, the translated products of the late tail genes of phages A118 and MC293/M188 are vastly dissimilar (Figure 4) despite their shared structural homologies with BppU and BppL of phage TP901-1. Phage A118 is another siphovirus belonging to Orthocluster IV of the *Listeria* phages, but differs from phages M188 and MC293 in that it displays strict host specificity for serovar 1/2 strains of *L. monocytogenes*, and cannot infect serovar 4 strains of the bacterium (Loessner et al., 2000). The lack of sequence conservation between these phages therefore may be as a result of their differing host specificities.

Overall, this research provides further insight into the complexity of phage-host interaction mechanisms in Gram-positive phages. Regarding *Listeria* phages in particular, the results of this study indicate that conserved baseplate architecture exists within the *Listeria* siphoviruses regardless of the *L. monocytogenes* host strain serotype, exemplified by the similarities observed between phages A118 and MC293/M188 in terms of TEM imaging and HHpred analysis. The similarities to the *Lactococcus* phage TP901-1 appear to indicate that this baseplate architecture may be conserved throughout the Gram-positive siphoviruses, though additional research is necessary in order to fully evaluate this theory. Further comparative analysis demonstrated that the late tail genes of phages M188 and MC293 are highly conserved throughout the serovar 4 specific *Listeria* phages, also indicating that conserved baseplate architecture may exist amongst this subgroup. The lack of sequence similarity in the late tail genes of phages A118 and MC293/M188 provides further evidence that the surface carbohydrate components of the *L. monocytogenes* cell represent the specific targets of *Listeria* siphoviruses, given that these cell surface components represent the only major compositional difference between the serovar 1/2 and serovar 4 cell walls. Finally, the lytic spectrum assessments coupled with the generation of spontaneous mutants together identify ORF 19 of phage M188 and ORF 20 of phage MC293 as the receptor binding proteins of these phages, and as such, this research ultimately adds to the current repository of knowledge regarding the RBPs of Gram-positive phages.

### Conflict of interest statement

The authors declare that the research was conducted in the absence of any commercial or financial relationships that could be construed as a potential conflict of interest.
